# Radiation damage to biological samples: still a pertinent issue

**DOI:** 10.1107/S1600577521008845

**Published:** 2021-09-01

**Authors:** Elspeth F. Garman, Martin Weik

**Affiliations:** a Department of Biochemistry, University of Oxford, South Parks Road, Oxford OX1 3QU, United Kingdom; b Univ. Grenoble Alpes, CEA, CNRS, Institut de Biologie Structurale, F-38044 Grenoble, France

**Keywords:** radiation damage, macromolecular crystallography, dose, serial crystallography, room-temperature crystallography, X-ray imaging, X-ray footprinting, SAXS, electron microscopy, XFEL simulations, single-wavelength anomalous dispersion

## Abstract

An understanding of radiation damage effects suffered by biological samples during structural analysis using both X-rays and electrons is pivotal to obtain reliable molecular models of imaged molecules. This special issue on radiation damage contains six papers reporting analyses of damage from a range of biophysical imaging techniques. For X-ray diffraction, an in-depth study of multi-crystal small-wedge data collection single-wavelength anomalous dispersion phasing protocols is presented, concluding that an absorbed dose of 5 MGy per crystal was optimal to allow reliable phasing. For small-angle X-ray scattering, experiments are reported that evaluate the efficacy of three radical scavengers using a protein designed to give a clear signature of damage in the form of a large conformational change upon the breakage of a di­sulfide bond. The use of X-rays to induce OH radicals from the radiolysis of water for X-ray footprinting are covered in two papers. In the first, new developments and the data collection pipeline at the NSLS-II high-throughput dedicated synchrotron beamline are described, and, in the second, the X-ray induced changes in three different proteins under aerobic and low-oxygen conditions are investigated and correlated with the absorbed dose. Studies in XFEL science are represented by a report on simulations of ultrafast dynamics in protic ionic liquids, and, lastly, a broad coverage of possible methods for dose efficiency improvement in modalities using electrons is presented. These papers, as well as a brief synopsis of some other relevant literature published since the last *Journal of Synchrotron Radiation* Special Issue on Radiation Damage in 2019, are summarized below.

## Abstract 2

An understanding of radiation damage effects suffered by biological samples during structural analysis using both X-rays and electrons is pivotal to obtain reliable molecular models of imaged molecules. This special issue on radiation damage contains six papers reporting analyses of damage from a range of biophysical imaging techniques.

Radiation damage remains one of the critical bottlenecks in the imaging of biological samples with X-ray and with electron beams, and manifests in both global degradation of signal with time and also as specific structural effects. X-ray sources used for diffraction measurements are producing ever higher flux densities, such as the recently commissioned Extremely Bright Source (EBS) at the ESRF in Grenoble. Moreover, the explosion in the number of cryo-electron microscopy molecular structure models now being deposited [*e.g.* see Fig. 4 in Assaiya *et al.* (2021 ▸)] has focused the attention of more researchers on the effects of radiation damage inflicted during the measurements.

There is already a substantial body of literature addressing various aspects of the radiation damage phenomena [see for example the 80 papers in special issues of the *Journal of Synchrotron Radiation* (*JSR*) arising from talks and posters given at the 2nd to 10th International Workshops on Radiation Damage to Biological Crystalline Samples, published in 2002, 2005, 2007, 2009, 2011, 2013, 2015, 2017 and 2019, respectively], which together provide a collected resource to researchers interested in the topic.

In this special issue of the *Journal of Synchrotron Radiation* there are six papers covering considerations of radiation damage effects and mitigation strategies in a wider range of biophysical imaging methods than previously presented in the above-mentioned collection of Workshop papers. These include aspects of sample degradation in macromolecular crystallography (MX), small-angle X-ray scattering (SAXS), X-ray footprinting mass spectrometry (XFMS) and cryo-electron microscopy (cryo-EM). In all cases, the focus is on finding strategies to mitigate the effects of absorbed dose (energy absorbed per unit mass, J kg^−1^ = gray, Gy). Dose is the preferred metric against which to monitor diffraction fading, increases in scaling *B*-factors, mosaicity and unit-cell parameters, as well as the specific structural damage suffered by the biological molecule under investigation, including heightened atomic *B*-factors in the final refined model. Typically the value of dose in an experiment cannot be measured, but can only be estimated from the particular beam conditions and sample constituents, so that reliable characterization of the required parameters (beam profile and flux) is important to be able to compare measurements taken under varying data collection protocols at different sites.

Collecting X-ray crystallographic data from tiny macromolecular crystals is becoming more and more widespread thanks to increasingly intense synchrotron microfocus beamlines (Yamamoto *et al.*, 2017 ▸), and is facilitated by dedicated programs, such as the automatic system for high-throughput structure analysis *ZOO* (Hirata *et al.*, 2019 ▸). The latter supports small-wedge synchrotron crystallography (SWSX) that consists of collecting small-wedge sub-datasets from a multitude of microcrystals mounted in a cryo-loop that are then merged into a full dataset. In this issue, Hirata and co-workers systematically address the effect of absorbed dose on SWSX structure determination (Baba *et al.*, 2021 ▸). In particular, they aimed to determine the optimal dose for sulfur-SAD (S-SAD) phasing. More than 400 small-wedge (10°) sub-datasets were collected at 100 K from densely packed lysozyme crystals (20 µm-sized) at five different doses (1, 2, 5, 10, 20 MGy per sub-dataset) and three different wavelengths (1.0, 1.4 and 1.7 Å), and the success of S-SAD phasing was monitored by calculating map correlation coefficients (CC_map_). At all three wavelengths, CC_map_ was highest at 5 MGy, implying that an optimal balance between diffraction intensity and radiation damage had been reached. This optimum-dose value is in line with the *point of diminishing returns* (about 3 MGy) for S-SAD phasing above which radiation-induced deterioration of data quality outweighs the gain in that data quality arising from increased multiplicity (Storm *et al.*, 2017 ▸).

Progress in observing and understanding X-ray radiation damage at 100 K has also been reported in some other publications since the last *JSR* radiation damage special issue. These include a re-examination of the resolution and dose dependence of global damage (manifested in reciprocal space) by Atakisi and co-workers (Atakisi *et al.*, 2019 ▸). Using datasets collected by other researchers from three different proteins, they suggest that these diffracted intensity decays can be explained by assuming a locally exponential dependence on dose, with a scattering-vector (*q*) dependence of *D*_1/2_ (the dose required to halve the diffraction intensity) of *D*_1/2_ (*q*) ≃ 1/*q* ^α^, where α ≃ 1.7. Factors affecting specific damage to particular amino acids (observed in electron density maps) have been investigated by Bhattacharyya and co-workers (Bhattacharyya *et al.*, 2020 ▸) who sought to explain the differential radiation sensitivity of di­sulfide bonds observed in six different proteins. They found that there was a correlation in the rate of damage and the proximity of the di­sulfide bond to carbonyl oxygen atoms which lie along the direction of the di­sulfide bond vector (Bhattacharyya *et al.*, 2020 ▸). They postulated that electron transfer is favoured along this route, resulting in a higher probability of di­sulfide bond damage than if there is no nearby aligned carboxyl oxygen. The experimental observations were complemented by density functional theory calculations using the B3LYP functional and a relatively flexible 6-31++G(2d,2p) basis set. Differential radiation sensitivity is also exhibited by the active sites in different monomers of a homotetrameric large-size subunit catalase (Zárate-Romero *et al.*, 2019 ▸) enzyme. This catalase causes disproportionation of hydrogen peroxide into molecular oxygen and two water molecules. The catalytic pathway involves the formation of an oxidized compound by reaction with the first H_2_O_2_ molecule, where the ferric resting state is converted to an oxyferryl intermediate. This is then reduced by a second H_2_O_2_ molecule back into the constituent heme in the ferric state. The reduction of the first compound formed (ferric to a previously unreported ferrous state) was monitored using both off-line (on solutions) and on-line UV-vis microspectrophotometry (on crystals) during X-ray irradiation, combined with structure determination at five absorbed doses. The researchers found that different members of the catalase tetramer were affected at varying rates (Zárate-Romero *et al.*, 2019 ▸), and suggest possible explanations for their observations. A robust and general explanation for the differential effects experienced by irradiated metals and various chemical groups is still lacking, and remains a challenge for MX radiation damage research.

Over the last few years, room-temperature (RT) crystallography has regained momentum at synchrotrons (Fischer, 2021 ▸), largely owing to the adoption of serial data collection strategies developed at X-ray free-electron lasers (XFELs). In serial synchrotron crystallography (SSX), radiation damage is reduced, but not eliminated, by distributing the total dose over thousands of microcrystals (Ebrahim, Moreno-Chicano *et al.*, 2019 ▸). The total dose per data set can be as low as 20 (Gotthard *et al.*, 2019 ▸), 11 (Ebrahim, Appleby *et al.*, 2019 ▸) or even 5 kGy (De la Mora *et al.*, 2020 ▸). To study global and specific radiation damage at RT, SSX has recently been used to collect a series of 40 data sets with finely sliced increasing dose increments at a dose rate of 40 MGy s^−1^ on hen egg-white lysozyme (HEWL) microcrystals (De la Mora *et al.*, 2020 ▸). Specific damage to disulfide bonds was observed, apparently running backward with dose above 0.5 MGy, a predicted effect (Holton, 2007 ▸) that can be explained by hole burning by the intense beam centre (Warkentin *et al.*, 2017 ▸). De la Mora *et al.* conclude that it is advisable not to exceed 0.38 MGy and 0.08 MGy in RT SSX experiments if global damage and specific damage to di­sulfide bonds are to be minimized, respectively.

From advances in both sample delivery and software methodology, the development of generally applicable serial femtosecond crystallography (SFX) data collection protocols has resulted in the collection of some notable damage-free structures of chromophore-containing proteins at XFEL sources over the last two years. These include a phytochrome photosensory module (GAF domain) which showed severe structural radiation damage to the chromophore in synchrotron cryo-MX structures and whose RT structure solution by SFX now paves the way for radiation-damage-free structure determination of photoconversion intermediates of that module (Burgie *et al.*, 2020 ▸). Another example is the SFX structure solution of a soluble methane monooxygenase hy­droxy­lase complex with its regulatory component in its fully oxidized and its fully reduced states (Srinivas *et al.*, 2020 ▸). Structure solution of this radiation-sensitive metalloprotein in its fully oxidized state has not been possible at synchrotron sources due to X-ray induced photoreduction. Srinivas *et al.* employed Fe *K*α X-ray emission spectroscopy concomitant with SFX data collection to identify the redox states of the oxidized (diferric) resting state and the chemically reduced (diferrous) state. Yet another example is the radiation-damage-free SFX structure determination of radiation-sensitive Compound II intermediates in two peroxidases (Kwon *et al.*, 2021 ▸). Fe—O bond lengths could be accurately determined and compared, including with those obtained from multi-crystal synchrotron and neutron structures. SFX has also been used to determine pristine structures of a Compound I intermediate in another peroxidase (Lučić *et al.*, 2020 ▸).

However, the potential for radiation damage artefacts in SFX structures needs to be understood, and progress towards this end has been made by the Schlichting group and colleagues. They carried out a systematic analysis of a number of damage indicators, including the length of the di­sulfide bonds in thaumatin (8) and a gadolinium derivative of lysozyme (4) as a function of the delay time between pairs of 15 fs-long XFEL pump and XFEL probe pulses with energies just below and just above the Fe *K*-edge at 7.112 keV, respectively. A thin iron foil over the front of the detector blocked the X-ray signal from the pump pulse. The range of time delay investigated in 15–20 fs steps was zero to 100 fs, during which time all the di­sulfide bond lengths increased approximately linearly from just above 2 Å to around 3.6 Å. The observed variation in bond length increase among the different bonds was attributed to the influence of their particular local environment within the protein. Simulations of the dynamics of the ions produced by the XFEL beam using two different methods were reported in the same paper and were able to reproduce the trends of the experimental data (Nass *et al.*, 2020 ▸). Thus, in order to obtain radiation-damage-free XFEL structures, the use of as short pulses as feasible is advisable.

In XFEL experiments, most of the doses thus far estimated in the literature do not represent the true situation in the experiment, since they do not include any time resolution. The diffraction process is much faster than most of the processes contributing to the dose, including that of photoelectron (PE) energy loss in the crystal. To address this challenge, the software program *RADDOSE-XFEL* has been developed to include a time stamp, and thus time resolution, for every interaction which contributes to the absorbed dose (Dickerson *et al.*, 2020 ▸). It has been suggested that damage would be observed in SFX structures when one ionization per atom had occurred, at a dose of around 400 MGy. This was calculated to be the dose at which the number of free electrons in an average protein equals the number of atoms by the end of an XFEL pulse (Chapman *et al.*, 2014 ▸). The time-resolved *RADDOSE-XFEL* simulations using all the relevant experimental parameters can be compared to this yardstick, in order to predict when damage might be expected to occur at XFEL sources. With the program, the authors investigated dose and also ionizations/atom as a function of pulse length, and, importantly, demonstrated that the time-resolved calculations for short pulses (<20 fs) resulted in doses that were over ten times lower than if the dose calculations were not time resolved (Dickerson *et al.*, 2020 ▸).

These recent experimental and computational reports on radiation damage to biological macromolecules at XFELs are complemented in this issue by the results of simulations of the ultrafast dynamics in ionic liquids initiated and probed by XFEL pulses (Patra *et al.*, 2021 ▸). The hybrid simulations which are reported capture ionization dynamics by plasma simulations and describe atomic motions by molecular dynamics simulations for a range of photon energies and beam intensities.

In terms of recent MX experiments to investigate the possibilities for extending crystal lifetimes, a study by Storm *et al.* tested the suggestion made by Nave and Hill 16 years ago (Nave & Hill, 2005 ▸) that, if the crystals were small enough, the primary PE would have a finite probability of exiting the crystal before it had lost all its energy, thus reducing the absorbed dose but retaining the same diffraction intensity (Storm *et al.*, 2020 ▸). At higher incident X-ray energy, the PEs are ejected with greater energy and thus have a bigger probability of escaping the crystal. Storm *et al.* used a PILATUS CdTe 2M detector and both small (5 µm × 3 µm × 3 µm) and larger (20 µm × 8 µm × 8 µm) cryo-cooled lysozyme crystals to collect a 5° wedge of data from over 100 crystals at incident energies of 13.5 and 20.1 keV. They found that *D*_1/2_ was higher both for small crystals and at higher energies. For the small crystals, *D*_1/2_ was 66% higher at 20.1 keV than at 13.5 keV if the dose calculations (Bury *et al.*, 2018 ▸) did not take PE escape into account, but agreed well if it was (Storm *et al.*, 2020 ▸). *RADDOSE-3D* (Bury *et al.*, 2018 ▸) can now compute both such doses. Complementing this experimental validation, recent Monte Carlo simulations which included the quantum efficiency characteristics of the Dectris CdTe detector concluded that an incident energy of 26 keV should be the optimum or ‘sweet spot’ to gain the maximum benefit from PE escape. The ‘diffraction efficiency’ (diffracted intensity/absorbed dose) was analysed for different crystal sizes and incident energies to judge the potential advantage of increased PE escape (Dickerson & Garman, 2021 ▸). It should be noted that taking advantage of this effect relies heavily on there being minimum buffer around the crystal (including at the front and back), as otherwise as many PEs can enter the sample from the irradiated buffer as can escape from the crystal.

SAXS measurements, usually carried out at RT, are now a versatile complementary tool for determining macromolecular envelopes [for a review see Brosey & Tainer (2019 ▸)] and the kinetics of protein–protein interactions. Several groups have previously investigated the use of radical scavengers to prolong the lifetime of samples in RT SAXS experiments. Recently, for instance, 5-methyl uridine, cytidine, cytosine and uridine were tested and found to increase the critical dose (defined variously by monitoring changes in scattered intensity and in the radii of gyration, as well as a criterion defined by the similarity of consecutive frames) by up to 20 times (Castellví *et al.*, 2020 ▸). In this issue, Stachowski *et al.* (2021 ▸) report such a study, but, unusually, they utilize for the measurements a protein which has been engineered to undergo a large-scale conformational change when a particular di­sulfide bond is reduced by the X-ray beam, making it an exquisitely sensitive signal for damage (Stachowski *et al.*, 2020 ▸). Sodium nitrate, cysteine and ascorbic acid at seven concentrations between 50 n*M* and 50 m*M* were tested for their efficacy in quenching di­sulfide bond breakage as shown by fits to the SAXS data. Sodium nitrate was found to be the most effective, inhibiting di­sulfide bond cleavage at 500 µ*M*, whereas it required 5 m*M* cysteine to achieve this. The optimum concentration of ascorbic acid was 5 m*M* but it could only inhibit fragmentation by around 75% after an absorbed dose of 792 Gy under the conditions employed. Dose estimation was carried out with *RADDOSE-3D* adapted for use with SAXS data collection protocols (Brooks-Bartlett *et al.*, 2017 ▸). Stachowski *et al.*’s results can be understood by considering the solvated electron rate constants for each scavenger, since their effectiveness mirrors this property. The authors point out that the engineered protein could also be useful for testing scavengers in MX by monitoring the di­sulfide radical anion signal at 400 nm with a UV-vis microspectrophotometer from solutions of protein with and without scavenger (Southworth-Davies & Garman, 2007 ▸). In MX a large range of small-molecule compounds have already been tested at both RT and 100 K, including the three used above (Barker *et al.*, 2009 ▸; Kmetko *et al.*, 2011 ▸; De la Mora *et al.*, 2011 ▸; Allan *et al.*, 2013 ▸), although the reported efficacy results show some discrepancies between different researchers.

Another protein characterization method that is now well established on at least two synchrotron beamlines but has not been described in the *JSR* Radiation Damage Special Issues before is that of X-ray footprinting (variously abbreviated to XF or XFMS). A broad-bandwidth (pink) X-ray beam is used to induce water radiolysis in dilute (1 to 10 µ*M*) aqueous RT samples of protein or nucleic acid dissolved in m*M* concentrations of various buffers, and the OH radicals (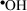) so produced react with solvent accessible sites, resulting in oxidative covalent modifications to the sample molecule. The fragments of the sample are then analysed by gel-electrophoresis or liquid chromatography mass spectrometry to identify the points of modification, thereby allowing the susceptible regions of the protein or nucleic acid to be inferred. The method can give information on the dynamics of macromolecular folding as well as on protein–protein and protein–ligand interactions, and its applicability and contributions to structural biology have recently been reviewed by Chance *et al.* (2020 ▸). To determine the X-ray dose, ‘dose response curves’ are collected prior to detailed data collection, but it should be noted that the dose in the XF field conventionally refers to the level of 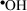 production rather than being the absorbed dose as in MX and SAXS. However, the number of 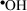
s available for footprinting depends critically on the scavenging properties of the buffer which is employed, so that similar absorbed doses can result in very different experimental outcomes. Typically, the sample solutions are oxygenated prior to irradiation, since dissolved molecular oxygen is necessary for most of the 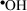-mediated modifications that are typically observed.

Two papers in this issue describe different aspects of XF data collection. Firstly Jain *et al.* present a detailed description of the new high-throughput XF beamline at NSLS II (XFP) which supplies X-rays to two endstations (Jain *et al.*, 2021 ▸). One of these can provide smaller (down to 120 µm × 450 µm) high-flux-density pink beams for irradiation of capillary flowed samples, producing very high 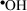 concentrations with microsecond X-ray exposures, even in samples which have intrinsic scavenging ability (*e.g.* membrane proteins, live cells). The second endstation supplies a defocused larger beam with lower flux density for irradiating smaller proteins. The various beamline components, sample delivery methodology and data collection protocols are covered in detail. Also described are experiments to probe the effect on the level of 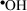 production when using 26 different reagents often used in sample buffers. As an indicator of 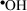 presence in these solutions, the authors use the Alexa488 fluoro­phore 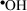 dose reporter molecule, whose fluorescence is destroyed by 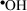 and so 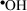 levels can be correlated to the utilized exposure time and X-ray flux (Gupta *et al.*, 2007 ▸). In the work reported here, the various buffers showed a range of responses. These results should allow better optimization of experimental practice, since the effects of the buffer on the irradiation necessary for successful outcomes is now more fully characterized.

As mentioned above, XF measurements are usually carried out on aerobic solutions in order to enhance the apparent 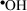 reaction rates. In the second contribution on XF to this special issue, Kristensen *et al.* describe experiments designed to investigate the effect of different oxygen concentrations on the extent of damage inflicted on three different protein solutions (cytochrome *c*, myoglobin and lysozyme) and compare this with the damage in aerated solutions (Kristensen *et al.*, 2021 ▸). They also tested these samples under more usual aerated conditions at different protein concentrations (2 or 5, 20 and 200 µ*M*) and found that a higher concentration decreased the oxidative modification rate, thus bestowing a protecting effect on the protein. At higher absorbed doses normally used in XF experiments, for both the aerated and low-oxygen content solutions they observed the formation of covalently linked higher molecular weight oligomers. The rate of side-chain modifications in the aerobic samples was higher than in those with low oxygen content, and some new modifications were observed in the latter samples. Notably, for the first time in XF, these authors estimate the absorbed doses in their experiments using *RADDOSE-3D*, as well as the more usual (for XF) ‘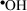 dose’.

Radiation damage effects are also an important consideration and have long been a concern in cryo-EM experiments (Henderson, 1995 ▸). Cryo-EM researchers have traditionally used electrons/unit area (Å^2^) as their ‘*x*-axis’ metric against which to plot radiation damage observables. However, a very recent paper has outlined the advantages of moving to the use of ‘dose’ in units of Gy for EM, and has detailed the necessary conversion factors for achieving this major change in the field (Egerton, 2021 ▸). It will thus be interesting to see if there is widespread adoption of this strong recommendation.

In the single-particle cryo-EM imaging of photosystem II at 1.95 Å resolution, observations of specific structural changes have been reported in regions affected by the radiation-induced alteration in redox states. The authors found they were able to minimize the damage by reducing the ‘dosage’ from to 50 to 2 frames (Kato *et al.*, 2021 ▸). In electron diffraction (ED) from microcrystals (µED) at 100 K, there has also been a demonstration of the use of specific structural damage to phase a seven amino acid peptide from a single zinc atom. This atom was located in a difference Patterson derived from two datasets, the first being collected with an average incident fluence of 0.17 e^−^Å^−2^, and the second after an exposure of 0.5 e^−^Å^−2^ (Martynowycz *et al.*, 2020 ▸).

In a paper in this special issue, Zhang *et al.* collect and summarize the possible options for improving the dose efficiency in single-particle cryo-EM, giving a comprehensive overview of the current status of their development. Five different modalities are described, explained and compared: laser phase plates, multi-pass transmission EM, off-axis holography, ptychography (a computational imaging technique) and quantum sorters, all of which may have the potential for increasing the signal obtained for a given dose. Experimental parameters discussed include illumination mode, beam energy, spatial resolution, electron fluence and sample thickness requirements, summarized in an informative table in the paper (Zhang *et al.*, 2021 ▸).

In conclusion, we should address the question of whether or not radiation damage to biological samples is still a pertinent issue in 2021. We believe it most certainly is, since in the near future even more researchers will come across the deleterious effects of the ever increasing X-ray flux densities and electron fluences that are being used to image their samples. Studies to understand different aspects of the phenomena have had a significant impact on all fields where ionizing radiation interacts with biological matter, and the community that has been established to investigate the effects has greatly improved the veracity of the biological knowledge gained. This impact is not likely to decrease in the future, and demonstrates the need for continuing work in the area. The broadening range of methods represented by the papers submitted to this special issue already reflects the heightened general interest of structural biologists in radiation damage effects. Since we sadly missed meeting in person during the 11th International Workshop on X-ray Radiation Damage to Biological samples held virtually at the Swiss Light Source, we are currently planning to hold the 12th Workshop in person there during 2022, pandemic conditions allowing.

*Note added in proof:* During preparation of this proof, a radiation damage comparison study was published on two small molecule catalysts which contain an iridium and a rhodium atom, respectively. Experimentally the authors used X-ray diffraction, X-ray powder diffraction and X-ray photoelectron spectroscopy (XPS) measurements to plot various radiation damage metrics against the absorbed dose. To our knowledge for the first time dose has been utilised for making XPS comparisons. DFT calculations were carried out to complement the observations (Fernando *et al.*, 2021 ▸).
